# What contextual factors and mechanisms facilitate male involvement in women's sexual and reproductive health in Sub-Saharan Africa? A rapid realist review protocol

**DOI:** 10.12688/hrbopenres.13113.2

**Published:** 2021-02-16

**Authors:** Purity Mwendwa, Caroline Karani, Elizabeth Kamolo, Thilo Kroll, Aoife De Brún, Eilish McAuliffe

**Affiliations:** 1 UCD Centre for Interdisciplinary Research Education and Innovation in Health Systems (UCD IRIS Centre), School of Nursing Midwifery and Health Systems, University College Dublin, Dublin, Ireland, Ireland; 2 School of Nursing, Meru University of Science and Technology, Meru, Kenya

**Keywords:** Sexual and Reproductive Health, Women's Health, Male Involvement, sub-Saharan Africa, Rapid Realist Review

## Abstract

**Background:**  Sexual and reproductive health (SRH) outcomes of women within low resource contexts continue to be of concern to policymakers. Notably, sub-Saharan Africa (SSA) continues to lag behind other regions of the world in improving SRH outcomes for women in the region. A key suggested strategy is male involvement through interventions that respect, promote and facilitate women in taking care of themselves and their new-borns. However, factors such as social-cultural barriers may preclude men's involvement in these programmes. There is a need for a context-specific understanding of gender dynamics and interaction and the mechanisms that enhance or impede men's involvement.

**Methods:** We will employ a rapid realist review (RRR) methodology to examine what mechanisms and contextual factors are essential to facilitate the involvement of men in women's SRH programmes in SSA. In keeping with the realist literature we will follow six steps, which will include: (1) developing a theory, (2) developing a search strategy, (3) selecting and appraising documents, (4) extracting data, (5) analysing data and synthesising the evidence, and (6) presenting and disseminating a revised theory. We will also engage with key stakeholders who will provide local contextual insights and with experts in the subject area. The review findings will be shared with relevant stakeholders using a variety of avenues including through publications, at conferences and on social media platforms.

**Discussion:** This review will identify the mechanisms and contextual factors that facilitate or hinder men's involvement in women's SRH programmes in SSA. The rationale for adopting an RRR approach is to help gather the information within a relatively short period to ensure relevance of findings to policymakers in SSA. Results from this work also have the potential to be adapted to the other contexts, for example, Ireland and the UK, which have a growing population of people from SSA.

## Introduction

Sexual and reproductive health (SRH) outcomes of women within low resource contexts continue to be a subject of concern ^1^. Although there has been a notable improvement in key health outcomes globally over the past two decades, sub-Saharan Africa (SSA) continues to lag behind other regions ^2^. For example, the region accounts for 66% of global maternal mortality ^2, 3^. This has been attributed to socio-economic and health system factors such as poverty, low literacy levels and limited health, human and physical infrastructure ^4^ as well as social-cultural barriers which preclude male involvement in women’s health ^5^. Male involvement in women's SRH has been recommended as a critical strategy for the improvement of health outcomes for this cohort. It is relevant to realising the global sustainable development goals 3 and 5 ^3, 6^. Male involvement in women's SRH is a broad term whose scope includes not only men's physical presence during women's reproductive care but also socio-economic and emotional support for women's health decision making ^7, 8^. This is based on the premise that, in most societies, men act as gatekeepers and primary decision-makers regarding resource utilisation and access to critical services, including reproductive health ^6^. Their decisions at all levels of society, both communal and at the basic family unit, can either impede or facilitate access to essential health services. This impacts on the health of women and girls.

Evidence suggests that despite challenges, male involvement in women’s health, particularly in low and middle-income countries, is yielding positive outcomes. For example, some programmes have reported increased adherence to ante-natal care attendance, birth readiness and delivery at a health facility where a male partner was involved ^9, 10^ while other programmes have shown an increase in the number of couples availing for HIV testing and those taking antiretroviral prophylaxis ^11^. However, challenges to male involvement have also been noted; despite their gatekeeping and decision-making roles, men have not been traditionally involved in women's health ^11^. Social-cultural barriers, such as societal constructions of masculinity, appear to prevent men from active involvement ^5^. Women and girls' sexual and reproductive health is mainly perceived as "female business", with men taking on the role of the provider of funds ^5, 12– 14^.

Furthermore, the accompaniment of women by their partners to health facilities is perceived as a form of emasculation, through crossing rigid lines of gender roles and norms, set by a highly patriarchal society ^5^. Other barriers to male partner involvement include economic barriers related to missed work opportunities due to accompaniment to health facilities as well as additional costs such as transport, more especially for men in the low-income bracket ^5, 13^. The perceived negative attitudes of staff at health facilities and in other cases, a lack of privacy at facilities, may preclude men's involvement ^5, 12, 13^. Limited knowledge among men on the importance of engaging in women's health as well as lack of interest in women's health are other notable barriers ^15^. Due to the vital role that men play in society within the SSA context, it is essential to further investigate critical drivers for facilitating their participation in women's SRH.

The need to facilitate men's involvement in women's SRH in SSA is well articulated in the literature. It includes several systematic reviews ^9, 10, 16^ that synthesis and assess available evidence to enhance and promote evidence-informed policymaking ^17^. However, systematic reviews fail to demonstrate how programmes work in diverse settings and within different populations ^18^, information that would be critical for informing policy decisions. Incorporating realist perspectives helps to elucidate why programs work or fail to work in specific contexts.

In this paper, we provide a protocol for a rapid realist review (RRR) that examines what contextual factors and mechanisms are essential in facilitating men to get involved in women's SRH in SSA. Realist research aims to provide explanations that clarify how interventions or programmes operate in specific contexts ^19^. Accordingly, because observations on their own cannot explain causal linkages between variables, it becomes necessary to demonstrate why relationships occur and to show what it is that leads to specific outcomes ^20^.

In realist reviews (RR), the Context + (plus) Mechanism = Outcome (CMO) heuristic tool forms the fundamental principles ^21^. Context denotes the history, culture, norms, beliefs, social networks as well as pre-existing structural organisations of the communities in which the interventions are conducted ^22, 23^. Mechanisms refer to the 'triggers' that lead participants to get involved or not in interventions and relates to their responses to the various intervention strategies and resources ^23^. Outcomes are the intended or unintended results based on the interplay between mechanisms and context ^19^. The outcome of an intervention, therefore, depends on particular decisions taken (or not) in regards to interventions and how actors reason about opportunities or resources availed by the intervention ^19^. The different components (CMO) are not static or linked in linear ways but dynamic and hence it is important to understand the dynamic interplay of the linkages between context, mechanisms and outcomes.

The main review objective is to examine what contextual factors and mechanisms play a role in facilitating or hindering men to get involved in women's SRH programmes in SSA.

*Specific objectives include*

1. To understand the different forms and types of male involvement in women's SRH
2. To identify contextual conditions and mechanisms that facilitate or impede men's involvement and develop an explanatory programme theory
3. To produce guidelines for consideration in the development of interventions to promote male involvement in women's SRH

## Methods

We will adopt the RRR methodology as it is best suited in contexts where evidence is limited and allows for the synthesis of knowledge in a considerably shorter time, compared to a traditional RR, making it possible to respond to time-sensitive policy decisions ^24^.

In realist reviews (RR), the context-mechanism-outcome (CMO) heuristic tool forms the fundamental principles intervention ^19^. For the current study, however, we use the expanded heuristic tool, intervention-context-actor-mechanism-outcome (ICAMO) ^25, 26^ that includes two additional components- ‘Intervention’ and ‘Actors’ based on the premise that interventions would need to be taken up by the Actors, if they are to succeed.

A critical strength of RRRs lies in the engagement of local reference groups and experts panels in the review process ^24^. Local reference groups contribute local contextual knowledge and include those individuals who are the target of the review findings, for example, policymakers, local community groups, the private sector, or charitable organisations. Findings from this review can inform the development of strategies to promote men’s involvement in women’s SRH programmes in SSA

Potential local reference groups will include key stakeholders in the Ministry of Health, Kenya at the national and county levels. Representatives will also be drawn from community-based organisations addressing SRH, community health workers and opinion leaders. The local reference group will share their knowledge and experience and help identify reports that can be included in the review and ultimately ensure that results have relevance for the local context ^24^. In preparation for this review, the first author, PM, has met with potential individuals and groups who will be part of the local reference panel. 

Expert panels include individuals knowledgeable in the content area. They are usually tasked with ensuring that the scope of the review remains focused and the process of searching for relevant literature is streamlined. In addition, they participate in the synthesis of findings while ensuring appropriate interpretation of the results ^24^. For the proposed review, the expert panel will consist of seven members with experience in women's health, methodologies that promote public involvement, nursing, public health, medical anthropology, psychology and health systems. We will employ a snowballing process to establish a panel of experts with experience in the field under study. In contrast, the local reference panel membership will be agreed by the expert panel ^27^. The time commitment required by the expert and reference groups will be kept to a minimum and highlighted in the invitation.

### Search strategy

In keeping with the realist literature, we will follow six steps in conducting the review ^28, 29^. This will entail: (1) developing a theory, (2) developing a search strategy, (3) selecting and appraising documents, (4) extracting data, (5) analysing data and synthesising the evidence, and (6) presenting and disseminating a revised theory.

Programme theories are statements that help to clarify how programmes or interventions are presumed to work ^30^. In realist evaluation, they form the units of analysis and serve to connect the (CMO) configurations ^28, 29^ and the theories become refined through testing. The initial programme theory will be developed based on a review of the literature and refined through expert panel and reference groups consultations to specify how men’s involvement in women’s SRH programmes could improve access and utilisation of services to improve women’s SRH.

Before the review commences, the expert panel will hold their first meeting to agree and clearly define the scope of the RRR, decide on terms to be included when searching the literature, and on the databases to be searched. The primary researcher (PM) will carry out an initial search of the literature to develop familiarity with the various male involvement strategies relevant to women's SRH in SSA. To search for relevant literature, the 'intervention', 'population' and 'context' will be included. The interventions to be studied include SRH programmes or initiatives, for example, family planning, ante-natal care and post-natal care programmes, and programmes for couples' counselling. The population of interest will be men (husbands, partners, spouses) involved in these interventions or programmes. The review will include studies located in SSA and conducted in any type of setting, including community, household, hospital or other health care facility settings. No restrictions will apply to research articles' study designs or to the year they were published. However, studies not addressing male involvement in women's SRH, not conducted in SSA and those in languages other than English will be excluded. We will also exclude commentaries, letters to editors and opinion pieces.

PM and ADB will undertake a search of the literature in consultation with a University faculty librarian. Databases are likely to include Web of Science, Pubmed, EMBASE, MEDLINE, and PsycInfo, based on other reviews ^31^ conducted in the SSA context. We anticipate that the literature for this topic will be diverse and hence we will use extensive searching of grey sources, such as OpenGrey, Google Scholar and DODRIA – Africa’s data directory – for relevant documents. Documents and articles, as identified by the local reference and expert panel members, will supplement the initial search. We will also search websites, such as those of the United Nations Children’s Fund (UNICEF), United Nations Population Fund (UNFPA) and the World Health Organization (WHO). The search will be iterative and refocused as the review evolves. PM and ADB will screen titles and abstracts for relevant literature.

### Data extraction

Data extracted will include information that helps identify contextual conditions and mechanisms that would facilitate male involvement in women’s SRH programmes. Such data would consist of i) the form and types of these programmes (family planning, ante-natal, post-natal), ii) pre-involvement activities such as communication campaigns (through media, mobile phones) sporting activities, the formation of men’s clubs, men’s health clinics, workshops, seminars, iii) settings where programmes are introduced, and iv) outcomes associated with these programs (for example increased ante-natal care attendance; reduced mortality and morbidity (mother and baby); decreased/increased intimate partner violence).

Two reviewers (CK and EK) will independently extract the data through a selection of text excerpts ^28^. We will use a modified version of the template for Intervention Description and Replication (TIDieR) to extract data ^32^. In the case of disagreements between the reviewers, consensus or engagement of a third reviewer will follow. The search for evidence and data extraction is expected to take between 12–14 weeks. The team will hold weekly data sessions to assess the review process. Extracted data will be reviewed for completeness by TK and EM.

### Analysing and Synthesising the evidence

Data analysis will incorporate both inductive and deductive approaches ^33^ and we will adopt Gilmore *et al.* approach ^34^ in the analysis and synthesis of the evidence. The Nvivo software will be used to support the management and analysis of the data. 

The experts, as mentioned earlier, and reference panels will scrutinise initial review findings, synthesis, examine and discuss the identified CMOs based on their experiences. Data will be synthesised to generate a 'programme theory' that aligns with the focus and scope of the review ^19^ and the 'programme theory' will be refined through group and individual discussions ^35^. This RRR will adhere to the realist publication standards guidelines, (RAMESES) ^29^, Realist And MEta-narrative Evidence Syntheses: Evolving Standards.

During the initial planning stages of this protocol, an advisory group working at the grassroots level in Kenya was set up. The group will have a representative at the local reference panel, and their input is expected to enhance the quality of the review process and the refining of the 'programme theory'. Importantly, the advisory team will play a critical role in supporting the dissemination of the review findings to policymakers and other knowledge users ^36^.

## Dissemination

The results that will emerge from the RRR will potentially be useful to policymakers and other key stakeholders, including NGOs and groups working to involve men in women's SRH programmes in SSA. The findings will also be presented to key policymakers, and relevant stakeholders and the aforementioned advisory group will be instrumental in enabling this process. We will also draft publications that will be submitted to high-impact, peer-reviewed journals, and the findings will be presented at academic conferences. We envision to present the results at the Africa Health Agenda International Conference. Also, an infographic will be developed based on the review findings and disseminated via social media platforms, for example, twitter, using various hashtags.

## Study status

Formation of the expert panel is complete. The searching of the literature has not commenced.

## Discussion

The planned RRR will synthesise and generate evidence on the contextual factors and mechanisms that enhance or hinder male involvement in women's SRH programmes. The findings will potentially have relevance to programmes that involve men, either as partners or spouses, or even key decision-makers. The RRR will provide knowledge synthesis within a short period, and to ensure that the evidence generated is relevant and suitable for the knowledge users, local reference panels and expert panels will guide the RRR. Involving these groups in the process not only facilitates the efficiency of identifying essential materials to include in the review, but has the potential to produce sufficiently robust findings which can inform current practice ^24^. We expect the review will have a political impact, influencing the development of national policy frameworks on male involvement in countries of SSA where such frameworks are lacking. The programme theories emerging from this work also have the potential to be adapted to the other contexts, for example, Ireland and the UK where there is a growing population from SSA.

## Data availability

No data is associated with this article.
