# Supplementary material for: What contextual factors and mechanisms facilitate male involvement in women's sexual and reproductive health in Sub-Saharan Africa? A rapid realist review protocol
Source: HRB Open Res. 2021 Feb 16;3:55. Originally published 2020 Aug 17. [Version 2] doi: 10.12688/hrbopenres.13113.2 (PMC7907758; doi:10.12688/hrbopenres.13113.2)
Supplement: Peer review history

# Peer review: Reviewer response for version 2

**DOI:** 10.21956/hrbopenres.14390.r28917

**Published:** 2021-02-25

**Authors:** Ferdinand Mukumbang

**Affiliations:** 1 Department of Global Health, University of Washington, Seattle, WA, USA

I am happy with the changes that the authors have provided to the reviewers' comments and queries.

Is the study design appropriate for the research question?

Yes

Is the rationale for, and objectives of, the study clearly described?

Yes

Are sufficient details of the methods provided to allow replication by others?

Partly

Are the datasets clearly presented in a useable and accessible format?

Not applicable

Reviewer Expertise:

I am a Health Policy and Systems Researcher in sub-Saharan Africa specialised in realist-informed methodologies.

I confirm that I have read this submission and believe that I have an appropriate level of expertise to confirm that it is of an acceptable scientific standard.

# Peer review: Reviewer response for version 2

**DOI:** 10.21956/hrbopenres.14390.r28916

**Published:** 2021-02-23

**Authors:** Katherine Semrau

**Affiliations:** 1 Harvard Medical School, Boston, MA, USA; 2 Ariadne Labs, Boston, MA, USA

Thank you for the revision and comments to the paper. I have no additional comments at this time.

Is the study design appropriate for the research question?

Yes

Is the rationale for, and objectives of, the study clearly described?

Yes

Are sufficient details of the methods provided to allow replication by others?

Partly

Are the datasets clearly presented in a useable and accessible format?

Not applicable

Reviewer Expertise:

I am a maternal and newborn health epidemiologist focused on design, testing and implementation of evidence-based interventions to improve quality of care and health outcomes.

I confirm that I have read this submission and believe that I have an appropriate level of expertise to confirm that it is of an acceptable scientific standard.

# Peer review: Reviewer response for version 1

**DOI:** 10.21956/hrbopenres.14219.r27833

**Published:** 2020-09-15

**Authors:** Katherine Semrau

**Affiliations:** 1 Harvard Medical School, Boston, MA, USA; 2 Ariadne Labs, Boston, MA, USA

Thank you for the opportunity to review the manuscript focused on a rapid realist review protocol for assessment of contextual factors and mechanisms of male involvement in SRH services in sub-Saharan Africa. The study protocol is clear and provides background to the approach of Dr. Mwendwa and colleagues. There are some key areas for clarification:

1. In the abstract, background, and discussion sections, the authors discuss sexual and reproductive health programs at a very general level. However, in the Methods section, there is specificity around targeting "strategies to address the practice of female genital mutilation/cutting (FGM/C)." Again, in the "Synthesing the evidence" section, the authors focus on anti-FGM/C experts to join the review panel. If the focus of the review and target audience for the findings is around FGM/C, then I would recommend including that throughout the study protocol paper. Further, that would modify the search terms/groupings for your search strategy. 
2. I agree with Dr. Mukumbang's comment that more information around data analysis would be beneficial, especially for others to replicate this approach.
3. Finally, as with all program implementation, there is the intention of a program approach, fidelity to the program approach or implementation plan, and then the success/failure of the program. Will the authors include programs that succeeded and failed with male involvement in SRH? It would be helpful to understand programs that failed as the contextual factors and mechanisms may have been important to the implementation.

Is the study design appropriate for the research question?

Yes

Is the rationale for, and objectives of, the study clearly described?

Yes

Are sufficient details of the methods provided to allow replication by others?

Partly

Are the datasets clearly presented in a useable and accessible format?

Not applicable

Reviewer Expertise:

I am a maternal and newborn health epidemiologist focused on design, testing and implementation of evidence-based interventions to improve quality of care and health outcomes.

I confirm that I have read this submission and believe that I have an appropriate level of expertise to confirm that it is of an acceptable scientific standard, however I have significant reservations, as outlined above.

# Response

**Published:** 2021-02-02

**Authors:** Purity Mwendwa

**Affiliations:** University College Dublin, Ireland

**Comments/queries 1**

 In the abstract, background, and discussion sections, the authors discuss sexual and reproductive health programs at a very general level. However, in the Methods section, there is specificity around targeting "strategies to address the practice of female genital mutilation/cutting (FGM/C)." Again, in the "Synthesing the evidence" section, the authors focus on anti-FGM/C experts to join the review panel. If the focus of the review and target audience for the findings is around FGM/C, then I would recommend including that throughout the study protocol paper. Further, that would modify the search terms/groupings for your search strategy.

**Response**

 The protocol has been amended throughout to reflect sexual and reproductive health programs more generally as opposed to making reference to female genital mutilation/cutting (FGM/C) programmes more specifically.

 The following changes have been effected as follows; **Methods, paragraph 4**

 Potential local reference groups will include key stakeholders in the Ministry of Health, Kenya at the national and county levels. Representatives will also be drawn from community-based organisations addressing SRH, community health workers and opinion leaders.

**Comments/queries 2**

 I agree with Dr. Mukumbang's comment that more information around data analysis would be beneficial, especially for others to replicate this approach.

**Response**

 This is now added to the section ‘ **Analysing and Synthesising** **the evidence’ paragraph 1**

 Data analysis will incorporate both inductive and deductive approaches (The RAMESES II Project, 2017) and we will adopt Gilmore et al (2019) approach in analysing and synthesising evidence. The Nvivo software will be used to support the management and analysis of the data.

**Comments/queries 3**

 Finally, as with all program implementation, there is the intention of a program approach, fidelity to the program approach or implementation plan, and then the success/failure of the program. Will the authors include programs that succeeded and failed with male involvement in SRH? It would be helpful to understand programs that failed as the contextual factors and mechanisms may have been important to the implementation.

**Response**

 The authors note that, while this would be an important aspect to include in the review, it is often difficult to identify literature in relation to failed interventions.

# Peer review: Reviewer response for version 1

**DOI:** 10.21956/hrbopenres.14219.r27835

**Published:** 2020-08-19

**Authors:** Ferdinand Mukumbang

**Affiliations:** 1 Department of Global Health, University of Washington, Seattle, WA, USA

Thank you for the opportunity to review this manuscript. In this manuscript, the authors propose a rapid realist review to understand how and why men's participation in sexual and reproductive health programmes can improve the health outcomes of women in SSA. I applaud the authors for proposing such an endeavour and I think that it is an important piece of work that is proposed. I have provided some comments and queries to help improve the quality of the submission.

 The authors write: "This has been attributed majorly to socio-economic and health system factors such as poverty, low literacy levels and limited health, human and physical infrastructure. Male involvement in women's SRH has been recommended as a critical strategy for the improvement of health outcomes for this cohort. It is relevant to realising the global sustainable development goals 3 and 5 ^3, 5^." I found it had to link male involvement as part of relevant interventions to improve SRH outcomes for women based on the socio-economic and health systems factors identified. I would be more inclined to male involvement to be related to socio-cultural barriers.

 The authors write: "However, systematic reviews fail to demonstrate how programmes work in diverse settings and within different populations ^18^ information that would be critical for informing policy decisions." I think that equally important especially in realist perspectives is to capture why other programs fail to work. 

 The authors write: "...the realist philosophy whose premise is to ascertain how programmes or interventions operate, or why they fall short, in particular environments and conditions ^20^." This statement is not entirely accurate. The epistemology of realist research is to provide mechanism-based causality explanations of social phenomena. Explaining how programmes or interventions work is the specific focus of realist evaluation, which is the focus of Pawson and Tilley.

 "Accordingly, because observations on their own cannot explain causal linkages between variables, it becomes necessary to demonstrate why relationships occur and to show what it is that leads to specific outcomes ^21^." I found this sentence confusing in the sense that I am not sure how it links the sentences before and after it. In other words, I am not sure how it establishes the realist evaluation's stance. It would have been important for the authors to establish why the notions of context, mechanisms and outcomes are central to realist evaluation.

 "In realist reviews (RR), the Context + (plus) Mechanism = Outcome (CMO) links form the fundamental principles ^23^". There seem to be some omissions in the above sentence. Consider replacing the word "links" with "heuristic tool".

 "In RRRs, the emphasis tends to be on the links between the interventions and outcomes,..." I am not sure what this means. RRRs are informed by the realist evaluation principles. Based on this notion, the statement highlight seems confusing to me.

 An explanation of what programme theories are will be useful. 

 "For the proposed study, we adopt the RRR methodology..." The first part of this sentence is redundant. Consider deleting. For the second part, it should be written as "We will adopt..."

 "As this review will inform the development of strategies to tackle the..." I am not so sure of the certainty that the authors display in this sentence but I will propose some modesty. Findings from this review can inform the development..."

 How is the initial programme theory going to the developed?

 Because there are various interventions that the authors are considering to implore, I would propose that they should consider using the intervention-context-actor-mechanism-outcome (ICAMO) heuristic tool towards their theory development. I have added a citation that illustrates the (ICAMO) heuristic tool and how useful it could be in review ^1^.

 There is no mention of how the data will be analysed. The process of data analyses should have been described as this is critical in how the initial programme theory translates to the refined programme theory and the linking or the CMO components towards theory development.

Is the study design appropriate for the research question?

Yes

Is the rationale for, and objectives of, the study clearly described?

Yes

Are sufficient details of the methods provided to allow replication by others?

Partly

Are the datasets clearly presented in a useable and accessible format?

Not applicable

Reviewer Expertise:

I am a Health Policy and Systems Researcher in sub-Saharan Africa specialised in realist-informed methodologies.

I confirm that I have read this submission and believe that I have an appropriate level of expertise to confirm that it is of an acceptable scientific standard, however I have significant reservations, as outlined above.

## References

[1] : Using the realist interview approach to maintain theoretical awareness in realist studies. *Qualitative Research*.2020;20(4) : 10.1177/1468794119881985 485-515 10.1177/1468794119881985

# Response

**Published:** 2021-02-02

**Authors:** Purity Mwendwa

**Affiliations:** University College Dublin, Ireland

**Comments/Queries 1**

 The authors write: "This has been attributed majorly to socio-economic and health system factors such as poverty, low literacy levels and limited health, human and physical infrastructure. Male involvement in women's SRH has been recommended as a critical strategy for the improvement of health outcomes for this cohort. It is relevant to realising the global sustainable development goals 3 and 53, 5." I found it had to link male involvement as part of relevant interventions to improve SRH outcomes for women based on the socio-economic and health systems factors identified. I would be more inclined to male involvement to be related to socio-cultural barriers.

**Response**

 We have amended the paragraph in the **introduction** as follows;

 ...This has been attributed to socioeconomic and health system factors (Girum,2017) as well as social-cultural barriers which preclude male involvement in women’s health (Ganle and Dery, 2017).

**Comments/Queries 2**

 The authors write: "However, systematic reviews fail to demonstrate how programmes work in diverse settings and within different populations18 information that would be critical for informing policy decisions." I think that equally important especially in realist perspectives is to capture why other programs fail to work.

**Response**

 We  have amended the paragraph in the **introduction 4 ^th^ paragraph**  as follows;

 However, systematic reviews fail to demonstrate how programmes work in diverse settings and within different populations (Davies,2006)  information that would be critical for informing policy decisions. Incorporating realist perspectives helps to elucidate why programmes work or fail to work in specific contexts.

**Comments/Queries 3**

 The authors write: "...the realist philosophy whose premise is to ascertain how programmes or interventions operate, or why they fall short, in particular environments and conditions 20." This statement is not entirely accurate. The epistemology of realist research is to provide mechanism-based causality explanations of social phenomena. Explaining how programmes or interventions work is the specific focus of realist evaluation, which is the focus of Pawson and Tilley.

**Response**

 We have corrected this  in the **introduction, 5 ^th^ paragraph** as follows;

 According to Pawson and Tilley, realist research aims to provide explanations that clarify how interventions or programmes operate in specific contexts (Pawson & Tilley, 1997).

**Comments/Queries 4**

 "Accordingly, because observations on their own cannot explain causal linkages between variables, it becomes necessary to demonstrate why relationships occur and to show what it is that leads to specific outcomes 21." I found this sentence confusing in the sense that I am not sure how it links the sentences before and after it. In other words, I am not sure how it establishes the realist evaluation's stance. It would have been important for the authors to establish why the notions of context, mechanisms and outcomes are central to realist evaluation.

**Response**

 This paragraph as been  restructured and the following sentence included: **Introduction paragraph 6**

 …The different components (CMO) are not static or linked in linear ways but dynamic and hence it is important to understand the dynamic interplay of the linkages between context, processes and outcomes.

**Comments/Queries 5**

 "In realist reviews (RR), the Context + (plus) Mechanism = Outcome (CMO) heuristic tool form the fundamental principles23". There seem to be some omissions in the above sentence. Consider replacing the word "links" with "heuristic tool".

**Response**

 The terms ‘heuristic tool’ have been added to this sentence

 In realist reviews (RR), the Context + (plus) Mechanism = Outcome (CMO) heuristic tool forms the fundamental principles.

**Comments/Queries 6**

 "In RRRs, the emphasis tends to be on the links between the interventions and outcomes,..." I am not sure what this means. RRRs are informed by the realist evaluation principles. Based on this notion, the statement highlight seems confusing to me.

**Response**

 This sentence has been omitted altogether

**Comments/Queries 7**

 An explanation of what programme theories are will be useful.

**Response**

 This  has been included under the section ‘ **search strategy’ paragraph 2**

 Programme theories are statements that help to clarify how programmes or interventions are presumed to work (Davidoff, Dixon-Woods, Leviton, & Michie, 2015).In realist evaluation, they form the units of analysis and serve to connect  the (CMO) configurations (Wong et al, 2013; Pawson and Tilley,1997) and become refined through testing

**Comments/Queries 8**

 "For the proposed study, we adopt the RRR methodology..." The first part of this sentence is redundant. Consider deleting. For the second part, it should be written as "We will adopt..."

**Response**

 This has now been amended within the **methods section**, **paragraph 1, the first sentence** to read;

 “We will adopt the RRR methodology…”

**Comments/Queries 9**

 "As this review will inform the development of strategies to tackle the..." I am not so sure of the certainty that the authors display in this sentence but I will propose some modesty. Findings from this review can inform the development..."

**Response**

 We have amended this in the **Methods, paragraph 3 as follows**;

 Findings from this review can inform the development of strategies to promote men’s involvement in women’s SRH programmes in SSA

**Comments/Queries 10**

 How is the initial programme theory going to the developed?

 We have added the following  paragraph under the section ‘ **search strategy’ paragraph 2 **as follows;

**Response**

 The initial programme theory will be developed based on a review of the literature and refined through expert panel and reference groups consultations to specify how men’s involvement in women’s SRH programmes could improve access and utilisation of services to improve women’s SRH.

**Comments/Queries 11**

 Because there are various interventions that the authors are considering to implore, I would propose that they should consider using the intervention-context-actor-mechanism-outcome (ICAMO) heuristic tool towards their theory development. I have added a citation that illustrates the (ICAMO) heuristic tool and how useful it could be.

**Response**

 We have taken this suggestion on board and amended this paragraph  in the **Methods, paragraph 3 **as follows;

 In realist reviews (RR), the context-mechanism-outcome (CMO) heuristic tool forms the fundamental principles (22) (Pawson, 1997). For the current study, however, we use the expanded heuristic tool, intervention-context-actor-mechanism-outcome (ICAMO)  proposed by (Marchal, Kegels, & Van Belle, 2018) and (Mukumbang, Marchal, Van Belle, & van Wyk, 2018)  that includes two additional components- ‘Intervention’ and  ‘Actors’ based on the premise that interventions would need to be taken up by the Actors if they are to succeed.  

**Comments/Queries 12**

 There is no mention of how the data will be analysed. The process of data analyses should have been described as this is critical in how the initial programme theory translates to the refined programme theory and the linking or the CMO components towards theory development.

**Response**

 This is now added to the section ‘ **Analysing and Synthesising** **the evidence’ paragraph 1 **as follows;

 Data analysis will incorporate both inductive and deductive approaches (The RAMESES II Project, 2017) and we will adopt Gilmore et al (2019) approach in analysing and synthesising evidence. The Nvivo software will be used to support the management and analysis of the data.
